# Resistin predicts disease severity and survival in patients with pulmonary arterial hypertension

**DOI:** 10.1186/s12931-024-02861-8

**Published:** 2024-06-06

**Authors:** Li Gao, John Skinner, Tanmay Nath, Qing Lin, Megan Griffiths, Rachel L. Damico, Michael W. Pauciulo, William C. Nichols, Paul M. Hassoun, Allen D. Everett, Roger A. Johns

**Affiliations:** 1 Department of Medicine, Division of Allergy and Clinical Immunology, Johns Hopkins University School of Medicine, 5501 Hopkins Bayview Circle, Room 3B.65B, Baltimore, MD 21224-6821 USA; 2 Department of Anesthesiology and Critical Care Medicine, Johns Hopkins University School of Medicine, 720 Rutland Avenue, Ross 361, Baltimore, MD 21287 USA; 3 Department of Biostatistics, Bloomberg School of Public Health, Johns Hopkins University, Baltimore, MD USA; 4 Department of Pediatrics, University of Texas Southwestern Medical Center, Dallas, TX USA; 5 Department of Medicine, Division of Pulmonary and Critical Care Medicine, Johns Hopkins University School of Medicine, Baltimore, MD USA; 6 Division of Human Genetics, Cincinnati Children’s Hospital Medical Center, Cincinnati, OH USA; 7 Department of Pediatrics, College of Medicine, University of Cincinnati, Cincinnati, OH USA; 8 Division of Pediatric Cardiology, Department of Pediatrics, Johns Hopkins University School of Medicine, Baltimore, MD USA

**Keywords:** Biomarker, Pulmonary arterial hypertension, Single nucleotide polymorphism, SNP, Machine learning, Resistin

## Abstract

**Background:**

Abnormal remodeling of distal pulmonary arteries in patients with pulmonary arterial hypertension (PAH) leads to progressively increased pulmonary vascular resistance, followed by right ventricular hypertrophy and failure. Despite considerable advancements in PAH treatment prognosis remains poor. We aim to evaluate the potential for using the cytokine resistin as a genetic and biological marker for disease severity and survival in a large cohort of patients with PAH.

**Methods:**

Biospecimens, clinical, and genetic data for 1121 adults with PAH, including 808 with idiopathic PAH (IPAH) and 313 with scleroderma-associated PAH (SSc-PAH), were obtained from a national repository. Serum resistin levels were measured by ELISA, and associations between resistin levels, clinical variables, and single nucleotide polymorphism genotypes were examined with multivariable regression models. Machine-learning (ML) algorithms were applied to develop and compare risk models for mortality prediction.

**Results:**

Resistin levels were significantly higher in all PAH samples and PAH subtype (IPAH and SSc-PAH) samples than in controls (*P* < .0001) and had significant discriminative abilities (AUCs of 0.84, 0.82, and 0.91, respectively; *P* < .001). High resistin levels (above 4.54 ng/mL) in PAH patients were associated with older age (*P* = .001), shorter 6-min walk distance (*P* = .001), and reduced cardiac performance (cardiac index, *P* = .016). Interestingly, mutant carriers of either rs3219175 or rs3745367 had higher resistin levels (adjusted *P* = .0001). High resistin levels in PAH patients were also associated with increased risk of death (hazard ratio: 2.6; 95% CI: 1.27–5.33; *P* < .0087). Comparisons of ML–derived survival models confirmed satisfactory prognostic value of the random forest model (AUC = 0.70, 95% CI: 0.62–0.79) for PAH.

**Conclusions:**

This work establishes the importance of resistin in the pathobiology of human PAH. In line with its function in rodent models, serum resistin represents a novel biomarker for PAH prognostication and may indicate a new therapeutic avenue. ML-derived survival models highlighted the importance of including resistin levels to improve performance. Future studies are needed to develop multi-marker assays that improve noninvasive risk stratification.

**Supplementary Information:**

The online version contains supplementary material available at 10.1186/s12931-024-02861-8.

## Introduction

Pulmonary arterial hypertension (PAH) is a multifactorial and life-threatening condition characterized by abnormal remodeling of distal pulmonary arteries. This remodeling leads to a progressive increase in pulmonary vascular resistance and subsequent right ventricular hypertrophy and failure [1]. Despite considerable advancements in PAH treatment over the past 30 years, prognosis remains poor [2]. One study that followed 162 consecutive patients with PAH reported that continuous treatment for at least 1 year with epoprostenol, the first therapy to be approved for the treatment of PAH, resulted in significantly greater survival rates at 1, 2 and 3 years of 87.8%, 76.3% and 62.8%, respectively, compared with expected survival rates (58.9%, 46.3%, and 35.4% based on historical data) [3, 4]. The phase 3 randomized controlled trial STELLAR demonstrated the clinical benefit of sotatercept, a TGFβ superfamily modulator, as an add-on treatment to stable background therapy for PAH [5]. Building on STELLAR findings, a recent study employed a population health model to assess the potential long-term clinical impact of sotatercept. According to this model, adding sotatercept to background therapy increased life expectancy by roughly three-fold among patients with PAH [6].

Mechanistic biomarkers, by serving as reliable predictors of PAH severity and survival, could be crucial for the development of novel treatment strategies. One potential mechanistic biomarker candidate is resistin, a member of the resistin-like molecule (RELM) family of pleiotropic cytokines [7]. Resistin, which was first identified as an adipokine in mice with insulin resistance properties [8], is predictive of poor clinical outcomes in patients with cardiovascular disease and heart failure [9–12]. RELM signaling is an important component of the type 2 inflammatory response to tissue injury in the lung and other organs [13, 14] and may be critically involved in inflammasome signaling and its downstream responses. We have shown that mRELMα, the mouse homolog of resistin, is dramatically upregulated in hypoxic lungs and produces potent mitogenic effects [15]. In rodent models, transtracheal delivery of mRELMα gene by adeno-associated virus causes vascular remodeling and hemodynamic changes like those of PAH [16]. Conversely, in vivo knockdown of mRELMα markedly reduces PAH development caused by chronic hypoxia or Th2 inflammatory stimuli [16–18], indicating an etiologic role for mRELMα in PAH. Human resistin is expressed by myeloid cells, especially macrophages [19]. Our mechanistic study of gene-modified mouse lines recently revealed that human resistin induces pulmonary vascular remodeling and PAH development by mediating the endothelial and smooth muscle cell crosstalk and macrophage activation dependent on activation of damage-associated molecular pattern (DAMP) signaling [20, 21]. Additionally, the cardiac-specific effects of human resistin on modulating inflammation in heart also have been revealed in our recent study [22].

We sought to assess the relationship of serum resistin levels with PAH disease severity and survival in a large cohort of patients with PAH composed mainly of two subtypes: idiopathic PAH (IPAH) and scleroderma-associated PAH (SSc-PAH). Because right ventricular hypertrophy and failure are the major causes of mortality in patients with PAH, we hypothesized that resistin levels would be associated with PAH severity (ie, hemodynamic measures) and mortality. Models combining resistin levels with clinical indicators have enhanced the ability to predict mortality compared with models that use clinical indicators alone. In this study, we investigate the role of resistin as a novel biochemical and genetic marker for PAH. Our findings will facilitate the development of precision prognostication tools and resistin-targeted therapy.

## Methods

### Study subjects

The National Biological Sample and Data Repository for PAH (also known as the PAH Biobank) is a National Institutes of Health–funded repository of biologic samples, clinical and genetic data collected from 36 enrolling PAH centers across North America. Biorepository data collection was approved by the institutional review board at each participating center, and all patients gave informed consent at the time of enrollment. Inclusion and exclusion criteria have been described elsewhere [23, 24], and details are in the online supplement. We received clinical data and biologic samples, including serum, from all patients who had IPAH or SSc-PAH and were 18 years of age or older (*n* = 1121). Samples from 50 healthy control subjects were obtained from Innovative Research to use as controls. This study was approved by the Johns Hopkins University Institutional Review Board.

### Measurement of serum resistin levels

ELISAs for resistin levels were successfully performed on serum from all patients. Briefly, serum resistin was analyzed in duplicate using the mesoscale discovery plate assay (Additional file 1: Supplementary Methods).

### Genotyping

DNA was extracted according to standard protocols. Genotyping for single nucleotide polymorphisms (SNPs) was carried out by using a genome-wide genotyping array (Illumina HumanOmni5, Illumina Inc., San Diego, CA, USA) and provided by the PAH Biobank, with an average completion rate of 98% [9]. Three SNPs within the gene that encodes resistin (*RETN*) and ~ 2 kb upstream (rs7408174 [T > C, upstream of *RETN*], rs3219175 [G > A, -2 kb variant], and rs3745367 [G > A, intron variant]) were covered by the array and analyzed for association with serum resistin levels.

### Statistical analyses

The chi-square test, Mann–Whitney U test, or Kruskal–Wallis test was used for comparisons between groups. To evaluate the performance of resistin level as a discriminator of PAH presence, we calculated the area under the curve (AUC) of the receiver operating characteristic (ROC) curve. Survival curves were computed with Kaplan–Meier estimates and the association between resistin level and survival was also tested with multivariable Cox regression models. Additionally, we used logistic regression models to test for genetic marker association with resistin levels as a qualitative phenotype. Age, sex, ethnicity, and BMI were included as covariates (Additional file 1: Supplementary Methods).

### Mortality model construction and assessment

Initially we selected 21 variables (Additional file 1: Supplementary Methods). These included demographics, clinical classification of PAH, and 10 hemodynamic measurements. Additionally, we included REVEAL 2.0 risk score, serum resistin levels, and the genotypes of three *RETN* SNPs (rs7408174, rs3219175, rs3745367). Then, we applied Lasso regression to the 13 quantitative variables. Lastly, we used 15 variables, including the 6 quantitative variables selected by Lasso, 4 additional hemodynamic variables, and 5 categorical variables, for the full model. For quality control processing of the data, 902 PAH patients (IPAH = 654) were enrolled after we removed subjects with missing values for quantitative variables. For machine-learning analysis, first, we randomly selected 70% (*n*= 631, IPAH = 455) of the patients as the training set for model construction. Next, we balanced the dataset using SMOTE-NC [25]. Then, five commonly adopted predictive model types were established to predict PAH mortality: random forest (RF), XGBoost, support vector machine (SVM), multilayer perceptron (MLP), and a stacking classifier. To obtain optimal prediction performance, K-folder cross validation (k = 5) was used to train, construct, and compare models. The confusion matrix, area under the ROC curve (AUC), sensitivity, positive predictive value (PPV), and F1 score (which is the harmonic mean of the sensitivity and PPV) were used to evaluate and compare the comprehensive performance of model types. Lastly, 30% of the entire cohort was included in the test set (*n* = 271, IPAH = 199) to validate the training set.

## Results

### Patient characteristics

Demographics and clinical characteristics of PAH patients in this study are presented in Table 1. The cohort was composed mainly of patients with IPAH (*n* = 808); the second largest disease subtype was SSc-PAH (*n*= 313). As in previous studies [24, 26], most patients were white women in the sixth decade of life, with New York Heart Association functional class (NYHA FC) III/IV symptoms; the median time from diagnosis to enrollment was approximately 4.8 years. Table 1 listed common comorbid conditions such as obesity (as determined by BMI), chronic renal disease, cardiovascular conditions (such as systemic hypertension and cardiomyopathy), and respiratory conditions (such as smoking, COPD and ILD/IPF). With the exception of ILD/IPF and renal insufficiency, which were more prevalent in SSc-PAH patients than in IPAH patients, co-morbidities were similar in patients with IPAH, SSc-PAH, and the entire cohort. Subjects had moderate to severe disease, with mean pulmonary artery pressure (mPAP) of 49 mm Hg (IQR: 19), pulmonary vascular resistance (PVR) of 8.95 Wood units (IQR: 7.03), and cardiac index of 2.54 L/min/m^2^ (IQR: 1.16). At enrollment, most patients were being treated with a phosphodiesterase-5 inhibitor or endothelin receptor antagonist therapy. The control cohort was 50% male and had a median age of 38 years (range: 18–57).

**Table 1:** Demographics and clinical characteristics of patients with pulmonary arterial hypertension

	**Overall (*n* = 1,121)**	**IPAH (*n* = 808)**	**SSc-PAH (*n* = 313)**
Age, median (IQR), y	58 (22)	53 (23)	65.5 (14)
Female sex, n (%)	919 (81.91)	645 (79.83)	274 (87.54)
Race, n (%)			
EA	947 (85.39)	676 (84.08)	271 (88.85)
AA	111 (10.00)	83 (10.32)	28 (9.18)
Other	51 (4.60)	45 (5.60)	6 (1.97)
Time from diagnosis to enrollment, mean ± SD, y	4.8 ± 4.66	5.41 ± 4.92	3.17 ± 3.39
Comorbidities, n (%)			
BMI, median (IQR), kg/m^2^	28.07 (9.69)	28.67 (10.51)	26.84 (7.79)
Cardiovascular			
Hypertension	397 (35.41)	276 (34.16)	121 (38.66)
Cardiomyopathy	20 (1.78)	13 (1.6)	7 (2.24)
Respiratory			
Smoker	461 (41.12)	335 (41.46)	126 (40.26)
Packs/per day, median (IQR)	1.0 (0.5)	1.0 (1.0)	1.0 (0.5)
Total no. of years, median (IQR)	19 (20)	18 (20)	20 (20)
COPD	106 (9.46)	81 (10.02)	25 (7.99)
ILD/IPF	84 (7.5)	14 (1.73)	70 (22.36)
Renal insufficiency	64 (5.71)	36 (4.46)	28 (8.95)
Deaths during follow-up, n (%)	191 (18.26)	96 (12.66)	95 (32.99)
6MWD, median (IQR), m	343 (164.75)	355 (155.75)	313.50 (169.5)
NYHA FC, n (%)			
I/II	299 (36.96)	203 (35.43)	96 (40.68)
III/IV	510 (63.04)	370 (64.57)	140 (59.32)
Hemodynamics, median (IQR)			
RAP, mm Hg	8 (7)	8 (7)	8 (8)
mPAP, mm Hg	49 (19)	52 (19)	43 (17)
PAWP, mm Hg	10 (6)	10 (6)	10 (6)
PVR, WU	8.95 (7.03)	10 (7)	7.31 (5.62)
CO, L/min	4 (1)	4 (2)	4 (2)
Cardiac index, L/min/m^2^	2.54 (1.16)	2.48 (1.17)	2.61 (2.07)
REVEAL Registry 2.0 risk score, median (range)	7 (13)	6 (13)	8 (11)
Biomarker values, median (IQR)			
NTproBNP, pg/mL	719 (2091)	490 (1428)	1796 (4434)
Resistin, ng/mL	6.63 (4.34)	6.2 (3.67)	8.28 (5.59)
Therapies, n (%)			
PDE5 inhibitor	794 (71.86)	568 (71.36)	226 (73.14)
ERA	626 (56.65)	455 (57.16)	171 (55.34)
IV/SC prostacyclin	297 (26.88)	249 (31.28)	48 (15.53)
CCB	125 (11.31)	98 (12.31)	27 (8.74)

*Abbreviations: 6MWD* 6-min walk distance, *AA* African American, *CCB* Calcium channel blocker, *CO* Cardiac output, *COPD* Chronic obstructive pulmonary disease, *EA* European American, *ERA* Endothelin receptor antagonist, *ILD/IPF* Interstitial lung disease/idiopathic pulmonary fibrosis, *IPAH* Idiopathic pulmonary arterial hypertension, *IV/SC* Intravenous or subcutaneous, *IQR* Interquartile range, *mPAP* Mean pulmonary arterial pressure, *NTproBNP* N-terminal pro–brain natriuretic peptide, *NYHA FC* New York Heart Association functional class, *PAWP* Pulmonary artery wedge pressure, *PDE5* Phosphodiesterase-5, *PVR* Pulmonary vascular resistance, *RAP* Right atrial pressure, *REVEAL Registry* Registry to Evaluate Early and Long-Term PAH Disease Management, *SSc-PAH* Scleroderma-associated pulmonary arterial hypertension, *WU* Wood units

### Serum resistin levels were significantly elevated in patients with PAH

**Fig. 1:**
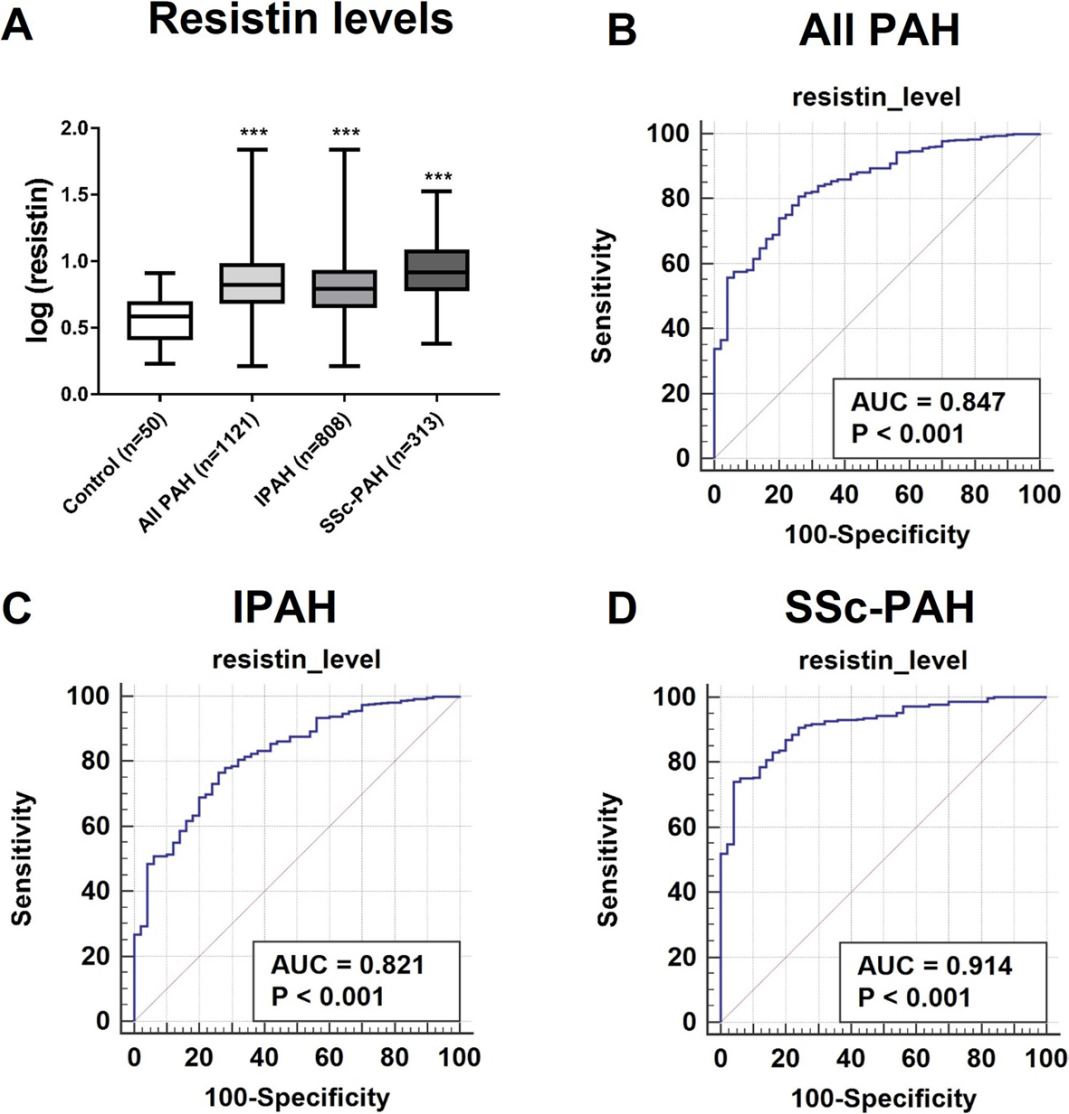
Comparison of serum resistin levels and receiver operating characteristic (ROC) curves. **A**, Resistin levels were significantly higher in patients with IPAH (*n* = 808, median [IQR] = 6.2 ng/mL [4.63–8.3]) and SSc-PAH (*n* = 313, 8.28 ng/mL [6.18–11.77]) than in controls (*n* = 50, 3.84 ng/mL [2.64–4.78]). ***, *P* < .0001, Kruskal–Wallis test. **B**–**D**, The specificity and sensitivity of serum resistin as a predictor for diagnosis of PAH in all PAH patients (*n* = 1121, **B**), IPAH patients (*n* = 808, **C**), and SSc-PAH patients (*n* = 313, **D**). *P* < .001 for all comparisons

Serum samples from 808 IPAH patients, 313 SSc-PAH patients, and 50 healthy control subjects (male-to-female ratio: 1:1) were available for analysis. When compared to circulating resistin levels in healthy control subjects (median [IQR] = 3.84 ng/mL [2.14]), levels were significantly higher in samples of the overall PAH cohort (*n* = 1121; 6.63 ng/mL [4.34]), in IPAH patients (6.2 ng/mL [3.67]), and in SSc-PAH patients (8.28 ng/mL [5.59]), all *P* < 0.0001 (Fig. 1A).

We used resistin levels from PAH patients and controls to generate an ROC curve for evaluating the specificity and sensitivity. Serum resistin discriminated all PAH, IPAH, or SSc-PAH from control subjects with AUCs of 0.85, 0.82, and 0.91 (Fig. 1B-D), respectively (*P* < 0.001). Based on this ROC curve analysis, we established a serum resistin threshold value of 4.54 ng/mL (defined by the Youden index) to distinguish healthy individuals from those with PAH or IPAH, and 6.30 ng/mL for SSc-PAH. Notably, we found no evidence that the control group's resistin levels were affected by sex. In the PAH cohort we examined, we are unable to completely rule out the chance that changes in sex or gender could affect the function of resistin. As such, in all relevant analyses, we considered sex as a potential confounding variable.

### Serum resistin levels were associated with metrics of PAH disease severity

Using multiple linear regressions in which we adjusted for age, sex, and BMI, we evaluated the relationship between serum resistin levels (log transformed) and continuous clinical variables, including invasive resting hemodynamics and exercise tolerance assessed by the 6-min walk distance (6MWD). In PAH patients, serum resistin was significantly associated with right atrial pressure (RAP, *P* < 0.026) and inversely associated with cardiac index (*P* < 0.014, Additional file 1: Table S1). Additionally, we observed a significant correlation between resistin levels and PAH severity measured by REVEAL Registry PAH risk score 2.0 (*P* < 0.001); each log-unit higher resistin was associated with a 0.1-point higher risk score. We observed similar trends for RAP (*P* < 0.032) and cardiac index (*P* < 0.01) for the IPAH subtype, but not in the SSc-PAH patients.

We further dichotomized PAH patients into resistin-level_low_ and resistin-level_high_ subgroups based on their serum resistin levels using a median split (Additional file 1: Supplementary Methods). As shown in Additional file 1: Tables S2 & S3, patients within the resistin-level_high_ group had shorter 6MWD (*P* = 0.001) and worse cardiac index (*P* = 0.016). Thus, PAH patients with higher resistin levels in the overall cohort had diminished functional capacity (NYHA FC III/IV vs. I/II*, P* = 0.014) and increased REVEAL Registry 2.0 risk score (*P* = 0.0001) that may contribute to the high mortality rate (23.1% vs. 13.4%, *P* = 0.0001).

### Serum resistin levels were associated with outcomes in PAH patients

#### Kaplan–Meier curves

We generated Kaplan–Meier curves to assess the relationship between elevated resistin levels and mortality. We arranged resistin levels in PAH patients (*n* = 1064) by quartiles: group 1 (< 25th percentile, *n* = 255; median log(resistin) = 0.609); group 2 (25th to 50th percentile, *n* = 233; median log(resistin) = 0.761); group 3 (50th to 75th percentile, *n* = 266; log(resistin) = 0.885); and group 4 (> 75th percentile, *n* = 241; median log(resistin) = 1.14). Figure S1 (Additional file 2) shows that survival was significantly shorter in subjects with higher resistin levels (chi-square = 23.5; *P* < 0.015 by log-rank test). A similar trend was observed for IPAH patients (chi-square = 10.94; *P* < 0.012 by log-rank test), but not for SSc-PAH patients.

#### Univariable Cox proportional hazard modeling

Given the strong predictive value of serum resistin for PAH outcome, we further constructed Cox proportional hazard models to examine this relationship. Resistin levels were significantly associated with mortality in univariable Cox proportional hazard modeling. A high resistin level (log transformed) was a significant predictor of adverse outcomes, with an unadjusted hazard ratio (HR) of 6.04 (95% CI: 3.20–11.39; *P*< 0.0001) in the overall cohort. Univariate analysis also showed associations between mortality and age, 6MWD, RAP, and mPAP, consistent with published data in other PAH cohorts [27–29]. When survival analyses were repeated in the two subgroups, the significance remained the same in IPAH (HR = 8.41; 95% CI: 3.41–20.71; *P* < 0.0001), but attenuated in SSc-PAH.

#### Multivariable Cox proportional hazards models

Multivariable models were built with adjustment for age, sex and BMI as well as hemodynamic variables associated with increased mortality in univariate analysis (mPAP and RAP). In multivariable Cox proportional hazards models (Table 2), the relationship between resistin levels and outcome remained significant in the overall cohort (HR, 2.6; 95% CI: 1.27–5.33; *P* < 0.0087) and in IPAH (HR, 3.29; 95% CI: 1.19–9.07; *P* < 0.0214) after adjusting for the following seven variables: age, gender, BMI, variables significant in the univariate analysis (RAP and mPAP) and NTproBNP. Furthermore, when multivariable analyses were repeated, excluding NTproBNP (reduced model 1) or only adjusting for age, sex and BMI (reduced model 2), the magnitude of biomarker associations with survival persisted in the overall cohort and IPAH.

**Table 2:** Multivariable Cox proportional hazard models for mortality

Predictor: (log) Resistin	Full model			Reduced model 1^a^			Reduced model 2^b^		
	**Hazard ratio**	**95% CI**	***P* value**	**Hazard ratio**	**95% CI**	***P* value**	**Hazard ratio**	**95% CI**	***P* value**
All PAH (*n* = 1,121)	2.60	1.27–5.33	**.0087**	3.43	1.71–6.89	**.0005**	3.73	1.89–7.36	**.0001**
IPAH (*n* = 808)	3.29	1.19–9.07	**.0214**	5.17	1.97–13.60	**.0009**	6.66	2.66–16.65	**.0001**
SSc-PAH (*n* = 313)	1.73	0.59–5.01	.311	2.05	0.71–5.92	.187	2.0	0.69–5.77	.20

The full model was adjusted for age, sex, BMI, right atrial pressure (RAP), mean pulmonary artery pressure (mPAP) and NTproBNP

^a^NTproBNP excluded from the full model

^b^The reduced model 2 was adjusted for age, sex and BMI

### RETN genetic variants were associated with serum resistin levels in PAH patients

We evaluated three *RETN* SNPs (rs7408174, rs3219175, and rs3745367) on the Omni5 Beadchip panel (Additional file 2: Figure S2A) for association with serum resistin level and clinical metrics for PAH severity. In 776 IPAH patients, two SNPs located in the proximal upstream (rs3219175) and intronic region (rs3745367) of *RETN* were associated with resistin levels. The coefficient r values were 0.218 (95% CI: 0.150–0.284; *P* = 0.0001) for rs3219175 and 0.134 for rs3745367 (95% CI: 0.065–0.203; *P* = 0.0002; Figure S2B). We further adjusted the models with age, sex, ethnicity, and BMI in logistic regression. In overall cohort of PAH patients, we observed significant adjusted *P* values of 0.0001 for both variants (Additional file 1: Table S4). In IPAH, with each additional copy of the AA or GA genotype for rs3219175, there was a 14.65-fold increased risk for having high resistin levels (above 6.20 ng/mL, tested under a dominant model). In contrast, the homozygous mutant carriers of rs3745367 (AA genotype, recessive model) had a 2.31-fold increased risk. However, no association signal was found for rs7408174. Similar trends were found for both variants in SSc-PAH patients (Additional file 1: Supplementary Results).

### Comparison of five mortality prediction models in the test set

**Fig. 2:**
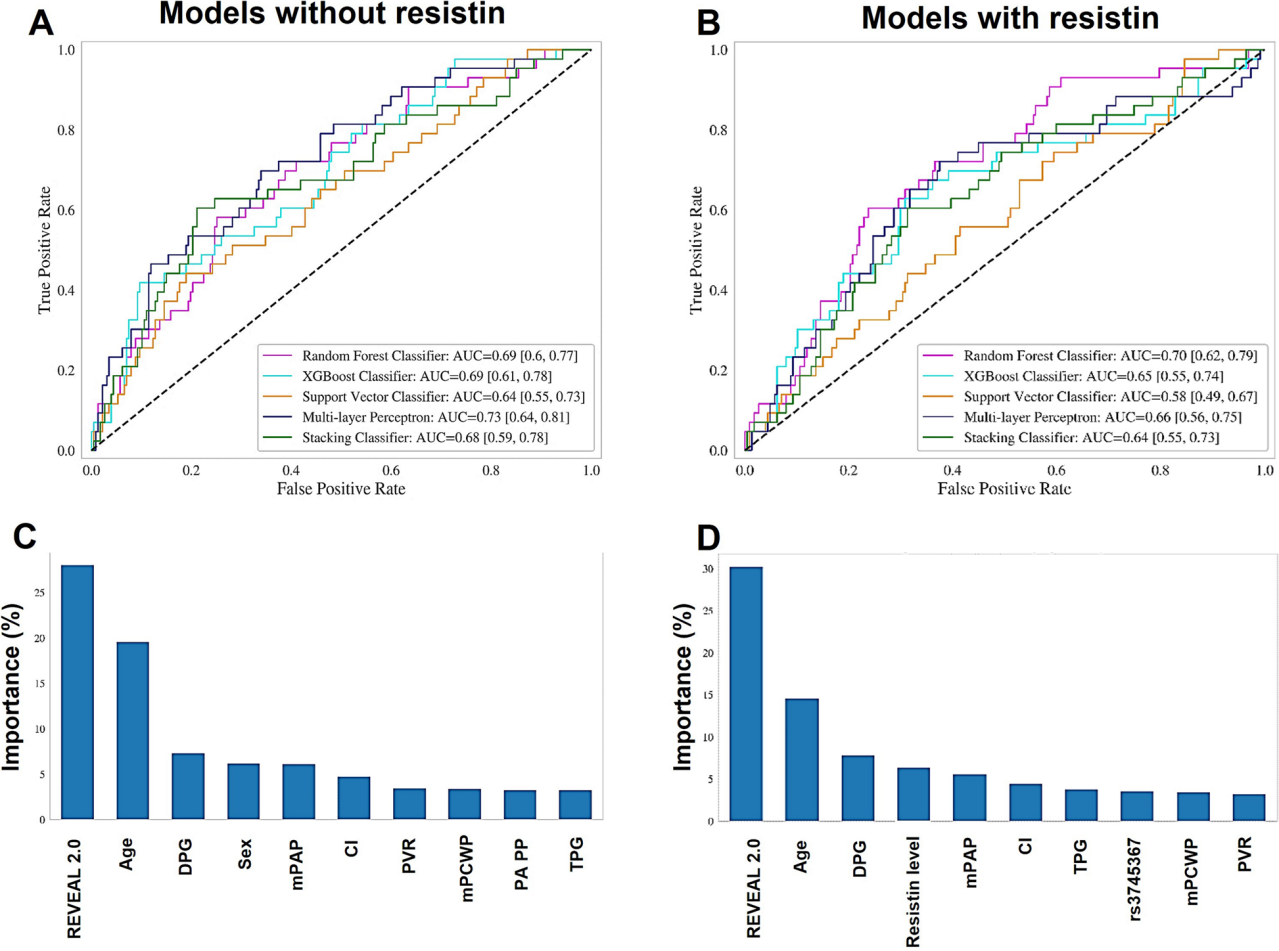
Evaluation of predictive models and analysis of the importance of each feature in classifying mortality. The ROC curves of the five models in the testing set were derived from selected parameters that excluded (**A**) or included (**B**) resistin levels and *RETN* gene SNPs. Mean AUC values and 95% CIs of different prediction models are shown. **C**, **D** Corresponding bar graphs describe the relative importance of the top 10 features in the random forest model. CI, cardiac index; DPG, diastolic pulmonary gradient; mPAP, mean pulmonary artery pressure; mPCWP, mean pulmonary capillary wedge pressure; PA PP, pulmonary arterial pulse pressure; PVR, pulmonary vascular resistance. TPG, transpulmonary pressure gradient

After confirming the association between high resistin levels and PAH severity and outcome, we sought to determine whether resistin, as a mechanistic biomarker, can improve the performance of mortality prediction models. First, we constructed models utilizing REVEAL 2.0 risk score, demographics (age and sex), clinical classification of PAH, and seven hemodynamic measurements. Five classifiers were established, and the average AUC and 95% CI for each classifier are shown in Fig. 2A. All five classifiers had AUC values above 0.60 (the acceptable cutoff for accuracy), and the MLP classifier obtained the highest AUC value of 0.73 (95% CI: 0.64–0.81). As shown in Additional file 1: Table S5, the five classifying models demonstrated varying performances for classifying non-survivors. The RF classifier was also the best-performing in the test set (AUC = 0.69; 95% CI: 0.60–0.77), with the highest sensitivity (0.58), precision (0.29), and F1 score (0.38). Second, we further constructed a full model (Fig. 2B) to include resistin levels and SNPs, in addition to the REVEAL 2.0 risk score and clinical variables mentioned above. Indeed, this full model (highest AUC = 0.70 from the RF classifier; 95% CI: 0.62–0.79) outperformed the model that excluded resistin levels and SNPs, exhibiting improved sensitivity (0.60), precision (0.29), and F1 score (0.39, Table S5). Then, we used the RF model to analyze the importance of features in predicting mortality. The 10 most important features are shown in Fig. 2C and D. REVEAL 2.0 risk score, age, diastolic pulmonary gradient [DPG], sex, and mPAP were the top 5 features that contributed to the model without resistin. In contrast, serum resistin level was among the top 5 important features in the full model. Of note, several other hemodynamic parameters (cardiac index, PVR, mean pulmonary capillary wedge pressure [mPCWP], and transpulmonary pressure gradient) also showed varying importance in models with and without resistin. Carrier status of *RETN* SNP rs3745367 was also among the top features in the full model.

## Discussion

Circulating resistin levels have an emerging role as biomarkers for a variety of diseases, including glucose metabolism and obesity [30, 31], diabetes [32], cancer [33], inflammatory diseases such as inflammatory bowel disease [34], and cardiovascular diseases [9, 10]. Because lung is the primary location of most RELM isoforms [7, 15, 35], research into the association between RELMs and the pathogenesis of cardiothoracic and respiratory diseases is now beginning to expand rapidly. In our study, we discovered that resistin levels were significantly higher in PAH patients as a group and in those with specific PAH subtypes, than they were in controls (*P* < 0.0001). Furthermore, when we used the AUC values of the ROC curve as criteria to evaluate how well resistin levels discerned the presence of PAH, all three tests had excellent discriminative ability (AUCs were 0.84, 0.82, and 0.91 for all PAH, IPAH, and SSc-PAH, respectively). More strikingly, when we further dichotomized IPAH patients into two subgroups using the identified threshold, we found that higher resistin levels were associated with worsening NYHA FC and reduced functional capacity. Additional evidence was found in survival analyses, supporting circulating resistin as a robust predictor of mortality in patients with PAH. The Kaplan–Meier curve analysis showed that elevated resistin levels (above the highest quartile) were significantly associated with increased risk of death in the overall cohort (*P* < 0.015 by log-rank test) and also in IPAH (*P* < 0.012). Of note, the relationship between resistin levels and outcomes persisted in multivariable Cox models in the overall cohort (*P* < 0.009), even after adjusting NTproBNP which is a known predictor for mortality in PAH. Thus, our study shows that serum resistin can serve as a biomarker for PAH prognosis and survival in a large cohort composed solely of patients with IPAH and SSc-PAH.

Resistin expression appears to be controlled in part by genetic programming, as genotypes of the *RETN*gene correlated with both level and disease state in some populations. Several SNPs have been shown to correlate with increased circulating resistin levels, and estimates suggest that approximately 70% of resistin expression can be attributed to genetic effects [36]. Gene variants in the promoter region upstream of *RETN*(− 420 C > G and − 638 G > A) appear to have the strongest effect. The − 420 C > G SNP (rs1862513, which was not covered by the Omni5 Beadchip) associated with increased circulating resistin levels has been associated with type 2 diabetes in several studies of Asian populations [37–39]. Additionally, the –420 C > G polymorphism was significantly associated with hypertrophic cardiomyopathy in a Pakistani population [40]. In our study, subjects who carried the minor allele of either the promoter variant rs3219175 or intronic variant rs3745367 had significantly higher resistin levels than did non-carriers; those with the promoter variant rs3219175 exhibited the strongest effects. Thus, our genetic analysis provides insight into the variation and complexity of resistin’s role in PAH.

The use of artificial intelligence in diagnosing respiratory diseases is rapidly evolving for prediction of sepsis, lung cancer prognosis, risk of hospital admission with chronic obstructive lung disease, and diagnosis of PAH [41–44]. To interpret the complex data for risk stratification in patients with PAH, we adapted common machine language techniques by training the algorithm on a cohort of 631 PAH patients (training data) to accurately predict PAH mortality. The reproducibility of the predictive performance quality was further verified on the test data composed of 271 PAH patients. The results indicated that RF classifier generated the best-performing predictive classifying model (obtained the highest predictive performance as indicated by an AUC value of 0.70) in the test set. Random forest is one of the ensemble models with advantages in handling mixed variable types, and it is robust to outlying observations. Based on the satisfactory predictive performance in RF models, we determined the relative importance of each attribute. Intriguingly, serum resistin levels ranked as the fourth most important feature after REVEAL 2.0 risk score, age, and DPG for predicting mortality in PAH patients. We utilized several hemodynamic parameters derived from the primary data including DPG (defined as diastolic PAP – mPCWP [mm Hg]). DPG previously has been reported to be associated with survival in group 1 pulmonary hypertension patients and portends poor prognosis in heart failure [45]. Another hemodynamic parameter, mPAP, also played an important role in the model, and recent evidence suggests that even mildly elevated mPAP is associated with morbidity and mortality. Therefore, in 2018, the hemodynamic definition of pulmonary hypertension was revised by lowering the threshold from mPAP ≥ 25 mmHg to > 20 mmHg [46]. Thus, our results clearly show that REVEAL 2.0 risk score is a robust predictor of mortality in PAH and that addition of resistin to survival models may improve model fit and predictive capacity.

The large sample size and complex clinical features within this cohort enabled important feature selection and extensive machine-learning–based multivariable modeling and model comparisons. To our knowledge, this study is among the very few to attempt machine-learning–based risk stratification in patients with PAH [47]. However, the study had several limitations. First, because this multicenter registry relies on separate reports from different centers for data collection, some covariates had missing data, notably 6MWD and NYHA FC. Despite the fact that 6MWD is a known predictor for PAH mortality and was also associated with increased mortality in univariate analysis, 6MWD was excluded due to significant missingness when we repeated the multivariable survival analyses (Table 2), which may affect the performance of predictive models. Second, some of the parameters included in the REVEAL Registry scoring tool for PAH risk prediction were unavailable in this cohort [48, 49]. Nevertheless, their omission is unlikely to have affected our results, as the REVEAL Registry risk score retains its predictive ability if at least seven of the 12 available risk parameters are available and included in the calculations. Third, most patients were receiving PAH-specific therapy at the time of biomarker assessment, which may have affected circulating biomarker levels. However, the association between resistin and mortality remained significant after adjusting for the presence and class of PAH therapy in multivariable models. Finally, serum collection was not contemporaneous with assessments of other clinical variables such as hemodynamics. Thus, performing the analyses in a subset of patients with biomarkers obtained within 6 months of other clinical measures of disease severity may strengthen the significance of biomarker associations with survival.

**Fig. 3:**
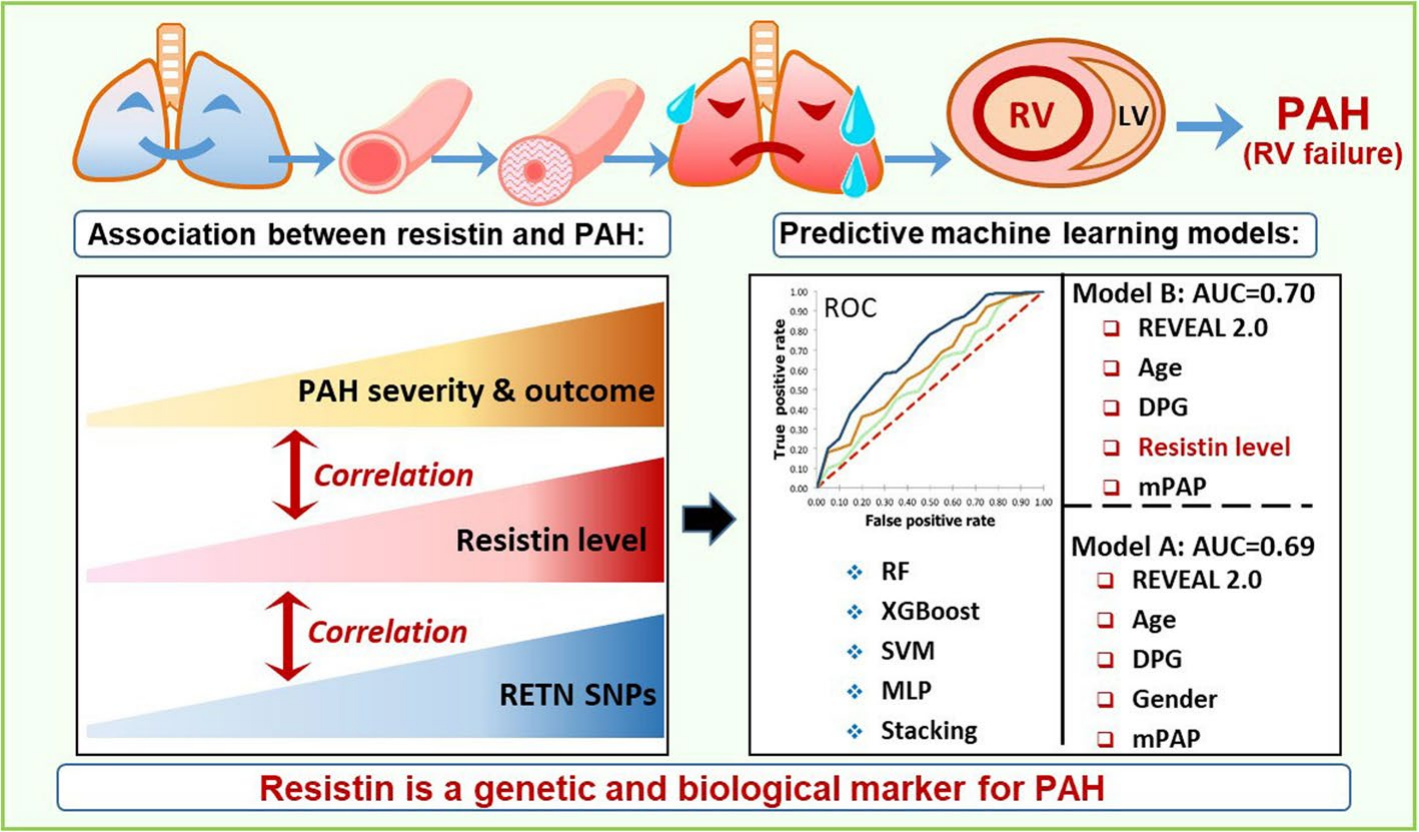
Role of resistin as a genetic and biological marker for PAH severity and adverse outcomes. Abbreviations: PAH, pulmonary arterial hypertension; RV, right ventricle; LV, left ventricle; RETN, gene that encodes resistin; SNP, single nucleotide polymorphism; ROC, receiver operating characteristic; AUC, area under the curve; RF, random forest; SVM, support vector machine; MLP, multilayer perceptron; mPAP, mean pulmonary artery pressure; DPG, diastolic pulmonary gradient; REVEAL 2.0, REVEAL 2.0 risk score

As illustrated in Fig. 3, our study provides evidence to support the use of circulating biomarkers as objective and accessible tools for noninvasive PAH risk stratification. Additional clinical, genetic, and epidemiologic studies are warranted to strengthen the association between resistin and the prevalence, severity, and outcome of PAH.

### Supplementary Information

Additional file 1. Supplementary Methods. Supplementary Results. Table S1. Correlation of log(resistin) Levels With Continuous Clinical Variables Adjusting for Age, Sex, and BMI. Table S2. Demographics and Clinical Characteristics of PAH Patients as a Function of Serum Resistin. Table S3. Demographics and Clinical Characteristics of Patients with SSc-PAH. Table S4. Association between Serum Resistin Levels and Genotypes of Two *RETN* Variants in PAH. Table S5. The efficacy of five machine-learning models for classifying non-survivors in the test set.

Additional file 2. Figure S1. The Kaplan-Meier mortality analysis of all PAH (*n*=998) and IPAH (*n*=722) patients by quartile of resistin levels. Group 1, <25^th^ percentile; group 2, 25^th^–50^th^ percentile; group 3, 50^th^–75^th^ percentile; group 4 (>75^th^ percentile). Figure S2. Two *RETN* single nucleotide polymorphisms (SNPs) are associated with resistin levels in IPAH patients (*n*=776). A, ENCODE regulation tracks on the *RETN* region (chromosome 19: 7,669,049–7,670,455). SNPs rs3219175 (located in proximal upstream) and rs3745367 (intronic region) are highlighted. B, Association between genotypes of the two *RETN* SNPs and resistin levels. *P*=.0001 for rs3219175 and .0003 for rs3745367.

## Data Availability

No datasets were generated or analysed during the current study.
