# Supplementary material for: Resistin predicts disease severity and survival in patients with pulmonary arterial hypertension
Source: Respir Res. 2024 Jun 6;25:235. doi: 10.1186/s12931-024-02861-8 (PMC11154998; doi:10.1186/s12931-024-02861-8)
Supplement: Supplementary file 1 — Additional file 1. Supplementary Methods. Supplementary Results. Table S1. Correlation of log(resistin) Levels With Continuous Clinical Variables Adjusting for Age, Sex, and BMI. Table S2. Demographics and Clinical Characteristics of PAH Patients as a Function of Serum Resistin. Table S3. Demographics and Clinical Characteristics of Patients with SSc-PAH. Table S4. Association between Serum Resistin Levels and Genotypes of Two RETN Variants in PAH. Table S5. The efficacy of five machine-learning models for classifying non-survivors in the test set. [file 12931_2024_2861_MOESM1_ESM.docx]

***Additional file 1***

Resistin Predicts Disease Severity and Survival in Patients with Pulmonary Arterial Hypertension

Li Gao, MD, PhD^1^*; John Skinner, BS^2^; Tanmay Nath, PhD^3^; Qing Lin, PhD^2^; Megan Griffiths, MD^4^; Rachel Damico, MD, PhD^5^; Michael W. Pauciulo, MBA^6,7^; William C. Nichols, PhD^6,7^; Paul M. Hassoun, MD^5^; Allen D. Everett, MD^8^; Roger A. Johns, MD^2,5^*

^1^Division of Allergy and Clinical Immunology, Department of Medicine, Johns Hopkins University School of Medicine, Baltimore, MD; ^2^Department of Anesthesiology and Critical Care Medicine, Johns Hopkins University School of Medicine, Baltimore, MD; ^3^Department of Biostatistics, Bloomberg School of Public Health, Johns Hopkins University, Baltimore, MD; ^4^Department of Pediatrics, University of Texas Southwestern Medical Center, Dallas, TX; ^5^Division of Pulmonary and Critical Care Medicine, Department of Medicine, Johns Hopkins University School of Medicine, Baltimore, MD; ^6^Division of Human Genetics, Cincinnati Children’s Hospital Medical Center, Cincinnati, OH; ^7^Department of Pediatrics, College of Medicine, University of Cincinnati, Cincinnati, OH. ^8^Division of Pediatric Cardiology, Department of Pediatrics, Johns Hopkins University School of Medicine, Baltimore, MD.

**Supplementary Methods**

**Measurement of Serum Resistin Levels**

Human resistin levels were determined by using a Meso Scale Discovery (MSD) R-PLEX human resistin assay (F21Z0/F21Z0-8, Meso Scale Diagnostics, Rockville, MD, USA). Samples were diluted 20-fold and run in duplicate. Plates were read on a MESO QuickPlex SQ 120 machine. Human resistin protein concentrations were determined by using MSD Workbench software. All concentrations were calculated on a 7-point calibration curve; the MSD software interpolates the optical density using a four-parameter logistic curve.

**Statistical Analyses**

Baseline characteristics are presented as median and interquartile range (IQR), number and percentage, or median and range, where appropriate. Resistin levels were not normally distributed; therefore, a nonparametric test was performed with log-transformed data. The chi-square test, Mann–Whitney U test, or Kruskal–Wallis test was used for comparisons between groups. Correlation analyses were carried out by using linear regression with adjustment for age, sex, and body mass index (BMI). To evaluate the performance of resistin level as a discriminator of PAH presence, we calculated the area under the curve (AUC) of the receiver operating characteristic (ROC) curve. Survival curves were computed with Kaplan-Meier estimates to determine time-to-death, and log-rank tests were performed to compare survival distributions. The association between resistin level and survival was also tested with multivariable Cox regression models. Additionally, we used logistic regression models to test for genetic marker association with resistin levels as a qualitative phenotype (high vs. low defined by a median split in each subgroup) and reported as an odds ratio with 95% confidence intervals. Age, sex, ethnicity, and BMI were included as covariates. A *P* value < .05 was considered statistically significant. Statistical analysis was carried out in MedCalc Statistical Software version 18.11.3 (MedCalc Software, Ostend, Belgium).

**Mortality model construction in the training set and assessment of five models in the test set**

We started by selecting 21 variables. These included demographics (age, sex, race, BMI, and body surface area); clinical classification of PAH; and hemodynamic measurements (mean pulmonary artery pressure [mPAP], right atrial pressure [RAP], mean pulmonary capillary wedge pressure [mPCWP], cardiac output [CO], pulmonary vascular resistance [PVR], PVR index [PVRI], cardiac index, pulmonary arterial [PA] pulse pressure, transpulmonary pressure gradient [TPG], and diastolic pulmonary gradient [DPG]). Additionally, we included REVEAL 2.0 risk score, serum resistin level, and the genotypes of three RETN SNPs (rs7408174, rs3219175, rs3745367). Then, we applied Lasso regression to the 13 quantitative variables for reduction of correlated inputs. Six variables (REVEAL 2.0 risk score, serum resistin level, age, mPAP, DPG, and pulmonary pulse pressure) had a corresponding Lasso coefficient that was non-zero. Finally, we used 15 variables, including the six quantitative variables selected by Lasso; four additional hemodynamic variables (RAP, PVR, mPCWP, and TPG); and five categorical variables (sex, clinical classification of PAH, and genotypes of the 3 SNPs) for the full model. For quality control, we first enrolled 902 PAH patients (IPAH=654) after removing subjects with missing values for quantitative variables. Then we randomly selected 70% (n=631) of the patients as the training set for model construction. Next, we balanced the dataset using the Synthetic Minority Oversampling Technique for Nominal and Continuous (SMOTE-NC).[^14^](#_ENREF_14) We established five commonly adopted predictive model types to predict PAH mortality: a random forest (RF) classifier model, an XGBoost model, a support vector machine (SVM), an artificial neural network of the multilayer perceptron (MLP), and a stacking classifier model. K-folder cross validation (k = 5) was used to train, construct, and compare the five predictive models. The confusion matrix, area under the ROC curve (AU-ROC), sensitivity (recall), positive predictive value (PPV [precision]), and F1 score (which is the harmonic mean of the precision and recall) were used to evaluate and compare the comprehensive performance of model types. Lastly, 271 patients (30% of the entire cohort), including 199 IPAH patients, were included in the test set to validate the training set.

**Supplementary Results**

**Serum Resistin Levels were Associated with Metrics of PAH Disease Severity**

We further dichotomized PAH patients into resistin-level_low_ and resistin-level_high_ subgroups based on whether their serum resistin levels were below or above the identified threshold (using a median split at 6.63 ng/mL). As shown in **Table S2**, patients within the resistin-level_high_ group were older (median [IQR]: 60 [23] vs. 55 [24] years, *P*=.001) and had shorter 6MWD (median [IQR]: 328 [167] vs. 564.5 [160] meters, *P*=.001), consistent with poor exertional tolerance. Additionally, resistin-level_high_ patients had worse invasive hemodynamic parameters, mainly cardiac index (median [IQR]: 2.51 [1.13] vs. 2.69 [1.13] L/min/m^2^, *P*=.016), and fewer received endothelin receptor antagonists (54.16% vs. 66.98%, *P*=.001). Thus, PAH patients with higher resistin levels had diminished functional capacity (NYHA FC III/IV vs. I/II*, P*=.014) and increased REVEAL Registry 2.0 risk score (*P*=.0001) that may contribute to the high mortality rate (23.1% vs. 13.4%, *P*=.0001). A similar trend was observed for IPAH: patients in the resistin-level_high_ group (above the threshold of 6.20 ng/mL) were older (median=54 [24] vs. 51.5 [23.25] years, *P*=.021) and had lower cardiac index (median [IQR]: 2 (1) vs. 3 (1) L/min/m^2^, *P*=.004), but had higher NTproBNP levels (501 [1434] vs. 472 [1396] pg/mL, *P*=.03). In contrast, when we stratified SSc-PAH patients into resistin-level_high_ and resistin-level_low_ groups, demographics and clinical characteristics were comparable between the two groups (**Table S3)**.

***RETN* Genetic Variants were Associated with Serum Resistin Levels in PAH Patients**

We evaluated three *RETN* SNPs (rs7408174, rs3219175, and rs3745367) on the Omni5 Beadchip panel (**Fig. S3A**) for association with serum resistin level and clinical metrics for PAH severity. In 776 IPAH patients, two SNPs located in the proximal upstream (rs3219175) and intronic region (rs3745367) of *RETN* were associated with resistin levels. The coefficient r values were 0.218 (95% CI: 0.150–0.284; *P*=.0001) for rs3219175 and 0.134 for rs3745367 (95% CI: 0.065–0.203; *P*=.0002; **Fig. S3B**). We further adjusted the models with age, sex, ethnicity, and BMI in logistic regression and observed significant adjusted *P* values of .0001 and .001, respectively (**Table S5**). Tested under a dominant model, homozygous and heterozygous mutant carriers of rs3219175 (AA and GA genotypes) had higher resistin levels (n=41, 11.69 ± 7.91 ng/mL) than did carriers of the wild-type GG genotype (n=735, 7.05 ± 4.70 ng/mL). With each additional copy of the AA or GA genotype, there was a 14.65-fold increased risk for having high resistin levels (above 6.20 ng/mL) in IPAH. Similarly, the homozygous mutant carriers of rs3745367 (AA genotype, recessive model) had higher resistin levels (n=89, 9.19 ± 5.73 ng/mL) than did the non-AA genotype carriers (n=688, 7.04 ± 4.87 ng/mL). However, no association signal was found for rs7408174.

In SSc-PAH patients, we observed significant adjusted values of 0.0007 for the promoter variant rs3219175 but only a nominal significant *P* value of .036 for the intronic variant rs3745367. However, both variants were associated with resistin levels in the overall cohort of PAH patients with an adjusted *P* value of .0001 (**Table S5**).

**Table S1.** Correlation of log(resistin) Levels With Continuous Clinical Variables Adjusting for Age, Sex, and BMI

| **Clinical variables** | **All PAH** | **IPAH** | **SSc-PAH** |
| --- | --- | --- | --- |
| 6MWD, m | -0.077 (-96.9 to 1.60; .058) | -0.062 (-104.973 to 22.096; .201) | -0.073 (-122.842 to 47.325; .382) |
| Hemodynamics |  |  |  |
| Heart rate, beats/min | 0.065 (1.014 to 23.225; **.032**) | 0.024 (-8.971 to 18.424; .498) | 0.155 (0.410 to 19.204; **.041**) |
| RAP, mm Hg | 0.067 (0.219 to 3.345; **.026**) | 0.076 (0.189 to 4.133; **.032**) | 0.062 (-1.342 to 4.366; .298) |
| mPAP, mm Hg | -0.005 (-4.135 to 3.419; .853) | 0.014 (-3.873 to 5.874; .687) | 0.067 (-2.491 to 9.205; .259) |
| PAWP, mm Hg | -0.002 (-1.18 to 1.096; .943) | 0.028 (-0.828 to 1.989; .419) | -0.040 (-2.836 to 1.408; .508) |
| PVR, WU | -0.006 (-1.853 to 1.497; .835) | -0.006 (-2.361 to 2.006; .873) | 0.077 (-0.854 to 4.096; .198) |
| CO, L/min | 0.015 (-0.510 to 0.852; .622) | 0.019 (-0.604 to 1.047; .598) | -0.015 (-0.928 to 0.708; .792) |
| Cardiac index, L/min/m^2^ | -0.075 (-0.696 to -0.079; **.014**) | -0.091 (-0.891 to -0.119; **.010**) | -0.065 (-0.689 to 0.199; .278) |
| REVEAL Registry 2.0 risk score | 0.101 (0.391 to 1.455; **.001**) | 0.031 (-0.419 to 1.110; .375) | 0.105 (-0.089 to 2.257; .070) |

All data are presented as regression coefficient (95% confidence interval; *P* value). Bold font indicates statistical significance.

Abbreviations: 6MWD, 6-minute walk distance; CO, cardiac output; IPAH, idiopathic pulmonary arterial hypertension; mPAP, mean pulmonary arterial pressure; PAWP, pulmonary artery wedge pressure; PAH, pulmonary arterial hypertension; PVR, pulmonary vascular resistance; RAP, right atrial pressure; REVEAL Registry, Registry to Evaluate Early and Long-Term PAH Disease Management; SSc-PAH, scleroderma-associated pulmonary arterial hypertension.

**Table S2.** Demographics and Clinical Characteristics of PAH Patients as a Function of Serum Resistin

| **Variables** | **All PAH** | | | **IPAH** | | |
| --- | --- | --- | --- | --- | --- | --- |
|  | **Resistin level_low_^a^ (n=219)** | **Resistin level_high_^b^ (n=903)** | ***P* Value^e^** | **Resistin level_low_^c^ (n=402)** | **Resistin level_high_^d^ (n=405)** | ***P* Value^e^** |
| Age, median (IQR), y | 55 (24) | 60 (23) | **.001** | 52 (24) | 54 (24) | **.01** |
| Female sex, n (%) | 450 (80.4) | 469 (83.5) | .178 | 315 (78.4) | 330 (81.5) | .039 |
| Race, n (%) |  |  | **.001** |  |  | .032 |
| EA | 462 (83.4) | 485 (87.4) |  | 332 (83.2) | 344 (85.1) |  |
| AA | 53 (9.6) | 58 (10.5) |  | 33 (8.3) | 50 (12.4) |  |
| Other | 39 (7) | 12 (2.2) |  | 34 (8.5) | 10 (2.5) |  |
| Deaths during follow-up, n (%) | 70 (13.4) | 121 (23.1) | **.0001** | 38 (10.1) | 58 (15.3) | **.032** |
| 6MWD, median (IQR), m | 364.5 (160) | 328 (167.25) | **.001** | 380 (155.25) | 343 (162.25) | .006 |
| NYHA FC, n (%) |  |  | **.014** |  |  | **.008** |
| I/II | 164 (41.2) | 135 (32.8) |  | 114 (40.7) | 88 (330.1) |  |
| III/IV | 234 (58.8) | 276 (67.2) |  | 166 (59.3) | 204 (69.9) |  |
| Hemodynamics, median (IQR) |  |  |  |  |  |  |
| RAP, mm Hg | 8 (6.75) | 8 (7) | .325 | 8 (7) | 8 (7) | .159 |
| mPAP, mm Hg | 50.5 (21) | 48 (19) | **.001** | 53 (19) | 51 (19.5) | .115 |
| PAWP, mm Hg | 10 (5) | 10 (6) | .072 | 10 (5) | 10 (6) | .209 |
| PVR, WU | 10 (8) | 9 (7) | **.006** | 10.02 (7.49) | 9.56 (7.56) | .228 |
| CO, L/min | 4 (2) | 4 (2) | .163 | 4.33 (1.73) | 4.40 (1.93) | .183 |
| Cardiac index, L/min/m^2^ | 2.75 (1) | 2.59 (1) | **.018** | 2.64 (1.17) | 2.37 (1.08) | **.002** |
| REVEAL Registry 2.0 risk score, median (range) | 6 (1-14) | 7 (1-13) | **.0001** | 6 (1-14) | 6 (1-13) | .181 |
| Biomarker value, median (IQR) |  |  |  |  |  |  |
| NTproBNP, pg/mL | 614 (1629) | 857.5 (2738) | **.001** | 438 (1435) | 546.5 (1374) | .582 |
| Therapies, n (%) |  |  |  |  |  |  |
| PDE5 inhibitor | 395 (71.9) | 399 (71.8) | .802 | 288 (73.3) | 280 (69.7) | .257 |
| ERA | 327 (59.6) | 299 (53.8) | **.052** | 241 (61.3) | 213 (53) | **.018** |
| IV/SC prostacyclin | 151 (27.5) | 146 (26.3) | .641 | 118 (30) | 130 (32.3) | .482 |
| CCB | 72 (13.1) | 53 (9.5) | .060 | 54 (13.7) | 44 (10.9) | .231 |
| Combination therapy, n (%) |  |  | **.034** |  |  | **.05** |
| Monotherapy | 138 (25.1) | 159 (28.6) |  | 89 (22.6) | 109 (27.1) |  |
| 2 drugs | 193 (35.2) | 213 (38.3) |  | 138 (35.1) | 149 (37.1) |  |
| ≥ 3 drugs | 218 (39. 7) | 184 (33.1) |  | 166 (42.2) | 144 (35.8) |  |

Abbreviations: 6MWD, 6-minute walk distance; AA, African American; CCB, calcium channel blocker; CO, cardiac output; EA, European American; ERA, endothelin receptor antagonist; IPAH, idiopathic pulmonary arterial hypertension; IQR, interquartile range; IV/SC, intravenous or subcutaneous; mPAP, mean pulmonary arterial pressure; NTproBNP, N-terminal pro–brain natriuretic peptide; NYHA FC, New York Heart Association functional class; PAH, pulmonary arterial hypertension; PAWP, pulmonary artery wedge pressure; PDE5, phosphodiesterase-5; PVR, pulmonary vascular resistance; RAP, right atrial pressure; REVEAL Registry, Registry to Evaluate Early and Long-Term PAH Disease Management; WU, Wood units.

^a^Resistin level_low_ = serum resistin level below 6.63 ng/mL cutoff for all PAH

^b^Resistin level_high_ = serum resistin level above 6.63 ng/mL cutoff for all PAH

^c^Resistin level_low_ = serum resistin level below 6.20 ng/mL cutoff for IPAH

^d^Resistin level_high_ = serum resistin level above 6.20 ng/mL cutoff for IPAH

^e^Chi-square test was used for categorical parameters, and nonparametric testing (Mann-Whitney) was performed for quantitative parameters. Bold font indicates statistical significance.

**Table S3.** Demographics and Clinical Characteristics of Patients with SSc-PAH

|  | **Resistin level_low_**^a^ **(n=156)** | **Resistin level_high_^b^ (n=157)** | ***P* Value^c^** |
| --- | --- | --- | --- |
| Demographics |  |  |  |
| Age, median (IQR), y | 63.5 (13) | 67 (13.25) | .01 |
| Female sex, n (%) | 131 (84) | 143 (91.1) | .057 |
| Race, n (%) |  |  | .35 |
| EA | 132 (87.4) | 139 (90.3) |  |
| AA | 15 (9.9) | 13 (8.4) |  |
| Other | 4 (2.6) | 2 (1.3) |  |
| Deaths during follow-up, n (%) | 47 (33.3) | 48 (32.9) | .934 |
| 6MWD, median (IQR), m | 304 (163) | 323 (183) | .689 |
| NYHA FC, n (%) |  |  | .538 |
| I/II | 48 (42.5) | 65 (57.5) |  |
| III/IV | 47 (38.5) | 75 (61.5) |  |
| Hemodynamics, median (IQR) |  |  |  |
| RAP, mm Hg | 8 (7) | 8 (7) | .306 |
| mPAP, mm Hg | 43 (15) | 43 (18.25) | .522 |
| PVR, WU | 7.35 (5.28) | 7.27 (6.57) | .836 |
| CO, L/min | 4.5 (1) | 4 (2) | .688 |
| Cardiac index, L/min/m^2^ | 2.63 (1.16) | 2.61 (0.95) | .695 |
| REVEAL Registry 2.0 risk score, median (range) | 8 (2-13) | 8 (2-13) | .828 |
| Laboratory chemistry, median (IQR) |  |  |  |
| NTproBNP, pg/mL | 1570.5 (3459) | 2022 (6045.5) | .239 |
| Therapies, n (%) |  |  |  |
| PDE5 inhibitor | 106 (69.3) | 119 (76.8) | .139 |
| ERA | 87 (56.9) | 83 (53.5) | .559 |
| IV/SC prostacyclin | 23 (15) | 25 (16.1) | .791 |
| CCB | 16 (10.5) | 11 (7.1) | .298 |
| Combination therapy, n (%) |  |  | .774 |
| Monotherapy | 50 (32.7) | 49 (31.6) |  |
| 2 drugs | 55 (35.9) | 63 (40.6) |  |
| ≥ 3 drugs | 48 (31.4) | 43 (27.7) |  |

Abbreviations: 6MWD, 6-minute walk distance; AA, African American; CCB, calcium channel blocker; CO, cardiac output; EA, European American; ERA, endothelin receptor antagonist; IQR, interquartile range; IV/SC, intravenous or subcutaneous; mPAP, mean pulmonary arterial pressure; NTproBNP, N-terminal pro–brain natriuretic peptide; NYHA FC, New York Heart Association functional class; PDE5, phosphodiesterase-5; PVR, pulmonary vascular resistance; RAP, right atrial pressure; REVEAL Registry, Registry to Evaluate Early and Long-Term PAH Disease Management; SSc-PAH, scleroderma-associated pulmonary arterial hypertension; WU, Wood units.

^a^Resistin level_low_ = serum resistin level below 8.28 ng/mL cutoff

^b^Resistin level_high_ = serum resistin level above 8.28 ng/mL cutoff

^c^Chi-square test was used for categorical parameters, and non-parametric testing (Mann-Whitney) was performed for quantitative parameters.

**Table S4.** Association between Serum Resistin Levels and Genotypes of Two *RETN* Variants in PAH

|  | **All PAH** | | | **IPAH** | | | **SSc-PAH** | | |
| --- | --- | --- | --- | --- | --- | --- | --- | --- | --- |
| ***RETN* variants** | **N** | **log (resistin)^a^** | ***P_adj_* (OR, 95% CI)^b^** | **N** | **log (resistin)^a^** | ***P_adj_* (OR, 95% CI)^b^** | **N** | **log (resistin)^a^** | ***P_adj_* (OR, 95% CI)^b^** |
| rs3219175 (G/A) | 1122 | 0.84 ± 0.22 |  | 808 | 0.81 ± 0.21 |  | 313 | 0.92 ± 0.22 |  |
| GG | 1015 | 0.83 ± 0.21 | .0001 (7.38, 3.47 – 15.68) | 735 | 0.79 ± 0.20 | .0001 (14.65, 5.17 – 41.53) | 280 | 0.91 ± 0.22 | .0007 (8.5, 1.73 - 41.9) |
| GA+AA | 56 | 1.01 ± 0.25 |  | 41 | 1.00 ± 0.24 |  | 15 | 1.03 ± 0.29 |  |
| rs3745367 (G/A) | 1122 | 0.84 ± 0.22 |  | 808 | 0.81 ± 0.21 |  | 313 | 0.92 ± 0.22 |  |
| GG+GA | 944 | 0.83 ± 0.21 | .0001 (2.53, 1.64 – 3.91) | 688 | 0.79 ± 0.20 | .001 (2.31, 1.39 – 3.85) | 256 | 0.92 ± 0.22 | .036 (2.38, 1.06 – 5.35) |
| AA | 128 | 0.91 ± 0.24 |  | 89 | 0.90 ± 0.24 |  | 39 | 0.94 ± 0.24 |  |

Abbreviations: OR, odds ratio; CI, confidence interval; PAH, pulmonary arterial hypertension; IPAH, idiopathic PAH; SSc-PAH, scleroderma-associated PAH.

**^a^**log (resistin) values are shown as mean ± standard deviation.

**^b^***P* values were adjusted for age, sex, ethnicity, and body mass index.

**Table S5.** The efficacy of five machine-learning models for classifying non-survivors in the test set^a^

| Model | **Recall**  **(Sensitivity, 95% CI)** | **Precision**  **(PPV, 95% CI)** | **F1-score**  **(95% CI)** |
| --- | --- | --- | --- |
| Model A |  |  |  |
| RFC | 0.58 (0.45, 0.71) | 0.29 (0.21, 0.37) | 0.38 (0.29, 0.47) |
| XGBC | 0.44 (0.32, 0.57) | 0.35 (0.24, 0.45) | 0.39 (0.28, 0.48) |
| SVC | 0.28 (0.17, 0.4) | 0.29 (0.18, 0.41) | 0.29(0.18, 0.39) |
| MLP | 0.58 (0.45, 0.71) | 0.27 (0.2, 0.35) | 0.37 (0.28, 0.46) |
| Stack | 0.21 (0.11, 0.32) | 0.31 (0.17, 0.46) | 0.25 (0.14, 0.36) |
| Model B |  |  |  |
| RFC | 0.60 (0.48, 0.73) | 0.29 (0.21, 0.37) | 0.39 (0.30, 0.48) |
| XGBC | 0.37 (0.25, 0.49) | 0.28 (0.18, 0.38) | 0.32 (0.22, 0.42) |
| SVC | 0.19 (0.09, 0.29) | 0.22 (0.11, 0.34) | 0.20(0.10, 0.30) |
| MLP | 0.53 (0.41, 0.67) | 0.28 (0.2, 0.36) | 0.37 (0.28, 0.46) |
| Stack | 0.12 (0.04, 0.2) | 0.22 (0.18, 0.37) | 0.15 (0.06, 0.25) |

^a^Model A utilized REVEAL 2.0 risk score, demographics, and hemodynamic measurements. Model B included serum resistin levels and SNPs in addition to features in model A.

Abbreviations: CI, confidence interval; MLP, multilayer perceptron; PPV, positive predictive value; RFC random forest classifier; stack, stacking classifier; SNP, single nucleotide polymorphisms; SVC, support vector classifier; XGBC, XGBoost classifier.
