# Supplementary material for: Resistin predicts disease severity and survival in patients with pulmonary arterial hypertension
Source: Respir Res. 2024 Jun 6;25:235. doi: 10.1186/s12931-024-02861-8 (PMC11154998; doi:10.1186/s12931-024-02861-8)
Supplement: Supplementary file 2 — Additional file 2. Figure S1. The Kaplan-Meier mortality analysis of all PAH (n=998) and IPAH (n=722) patients by quartile of resistin levels. Group 1, <25th percentile; group 2, 25th–50th percentile; group 3, 50th–75th percentile; group 4 (>75th percentile). Figure S2. Two RETN single nucleotide polymorphisms (SNPs) are associated with resistin levels in IPAH patients (n=776). A, ENCODE regulation tracks on the RETN region (chromosome 19: 7,669,049–7,670,455). SNPs rs3219175 (located in proximal upstream) and rs3745367 (intronic region) are highlighted. B, Association between genotypes of the two RETN SNPs and resistin levels. P=.0001 for rs3219175 and .0003 for rs3745367. [file 12931_2024_2861_MOESM2_ESM.pptx]

## Slide 1
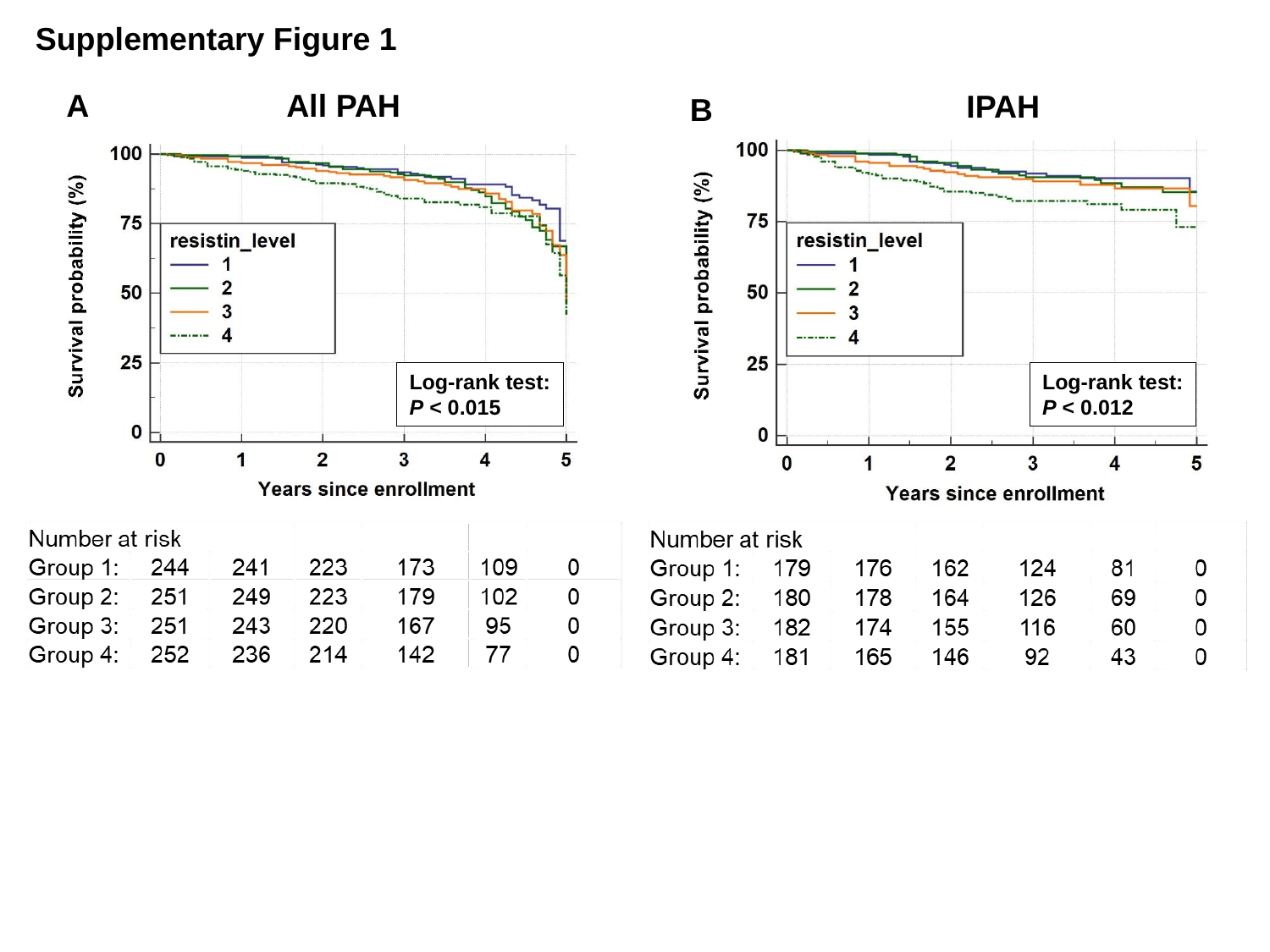

Supplementary Figure 1
A
All PAH
Log-rank test:
P < 0.015
IPAH
Log-rank test:
P < 0.012
B

## Slide 2
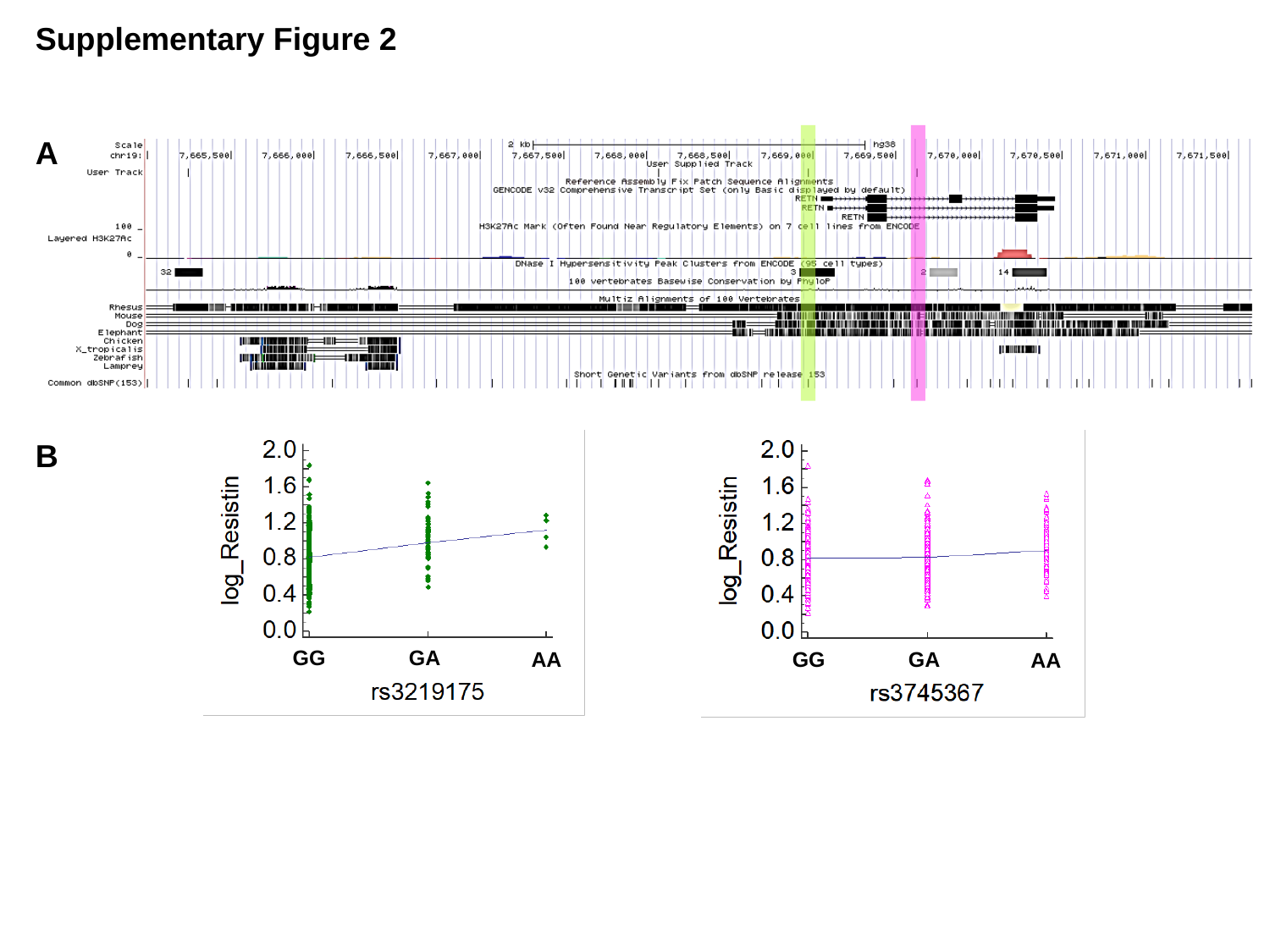

Supplementary Figure 2
A
B
GG
GA
AA
GG
GA
AA
